# The association between plant and animal protein intake and quality of life in patients undergoing hemodialysis

**DOI:** 10.3389/fnut.2023.1219976

**Published:** 2023-09-19

**Authors:** Melika Darzi, Mohammad Hossein Rouhani, Seyed-Ali Keshavarz

**Affiliations:** ^1^Department of Nutrition, Science and Research Branch, Islamic Azad University, Tehran, Iran; ^2^Nutrition and Food Security Research Center, Isfahan University of Medical Sciences, Isfahan, Iran

**Keywords:** dietary protein, animal protein, plant protein, quality of life, hemodialysis

## Abstract

**Background:**

Hemodialysis (HD) patients often experience a significant reduction in quality of life (QOL). The source of dietary protein intake may influence the renal function and complications of HD patients. The present study assessed the relationship between plant and animal protein intake and QOL in HD patients.

**Methods:**

264 adult patients under dialysis for at least three months were included in this cross-sectional study. Dietary intakes were collected using a valid and reliable 168-item semi-quantitative food frequency questionnaire (FFQ) over the past year. Total, animal, and plant proteins were calculated for each patient. To evaluate QOL, Kidney Disease Quality of Life Short Form (KDQOL-SF 1/3) was used. Anthropometric measures were assessed according to standard protocols.

**Results:**

In this study, the average age of participants was 58.62 ± 15.26 years old; most (73.5%) were men. The mean of total, plant, and animal proteins intake were 66.40 ± 34.29 g/d, 34.60 ± 18.24 g/d, and 31.80 ± 22.21 g/d. Furthermore, the mean score of QOL was 59.29 ± 18.68. After adjustment for potential confounders, a significant positive association was found between total dietary protein intake and QOL (*β* = 0.12; *p* = 0.03). Moreover, there was a significant association between plant-based protein intake and QOL (*β* = 0.26; *p* < 0.001). However, the association between animal protein intake and QOL was insignificant (*β* = 0.03; *p* = 0.60).

**Conclusion:**

Higher total and plant proteins intake were associated with better QOL in HD patients. Further studies, particularly prospective ones, are needed to corroborate these associations.

## Introduction

End-stage renal disease (ESRD) is the final and irreversible stage of chronic kidney disease (CKD) (1). The prevalence of ESRD is estimated to range between 8 and 16% worldwide (2) and is increasing annually. Hemodialysis (HD) is the primary and most prevalent treatment for ESRD patients (1). According to the International Society of Nephrology, approximately 2.5 million people are undergoing dialysis treatment (2). Unfortunately, despite advancements in dialysis treatment and medications, the mortality rate for these patients is still high (3). HD patients may experience complications and symptoms such as constipation, nausea, fatigue, pruritus, sleep disturbances, and depression that can reduce their ability to perform daily activities independently and ultimately reduce their quality of life (QOL) (4). QOL refers to a person’s satisfaction with their life concerning their expectations, goals, relationships, and independence, consisting of mental and physical well-being (5). In addition, QOL is an essential clinical measure that demonstrates the efficacy of health care and can be used to predict patient mortality (6). Studies revealed that HD patients have a lower QOL than the general population (7).

Diet and nutritional status play critical roles in HD complications and QOL (8). Protein intake is one of the most important dietary factors for these patients (9). Inadequate protein intake can lead to malnutrition, especially in elderly patients (6). Furthermore, it can increase the risk of inflammation and worsen the HD patient’s complications, directly impacting the patients’ physical and mental well-being (7). However, on the other side, high protein consumption elevates levels of urea and creatinine, which increase uremic symptoms in HD patients (10). According to the Kidney Disease Outcomes Quality Initiative (KDOQI) guideline, the recommended dietary protein intake during HD is 1.0–1.2 g/kg-day to maintain a stable nutritional status (11).

Moreover, recent studies have investigated that protein sources (plant or animal) may potentially impact kidney function and complications in HD patients (12, 13). They are advised to consume limited plant-based protein to help control their serum phosphorus and potassium levels (14). Some guidelines recommend that at least half of their dietary protein should come from animal sources to ensure they get enough essential amino acids (15, 16). Animal-based protein have a higher biological value than plant-based protein (17). However, several studies have shown that plant-based diets and higher intakes of plant-based protein have positive effects on patients’ status (18–20). A cohort study revealed that higher intake of vegetables and fruits in HD patients was associated with lower mortality (21). Another longitudinal cohort study indicated adherence to a plant-based diet in these patients was not related to hyperkalemia and appeared to be associated with improved nutritional status. In this study, the mean of total, plant, and animal proteins intake were 59.13 g/d, 21/8 g/d, and 37.33 g/d (22). Furthermore, recent studies have found that a plant-based diet is not associated with malnutrition in HD patients, and higher intake of plant-based protein may provide adequate quantity and quality, especially when consumed from diverse sources (23, 24). Additionally, plant-based proteins decrease acidosis, whereas animal-based proteins increase the acid load and can cause hyperfiltration and proteinuria (25, 26). Moreover, plant-based proteins are rich in phytochemicals and antioxidants, while red meat and processed meat are high in saturated fatty acids (SFA) and sodium, which can increase inflammation and exacerbate patients’ complications (27).

Previous studies evaluated the association between dietary protein sources and CKD patients’ risk, progression, and mortality. Nevertheless, to our knowledge, no study has been conducted to examine the relationship between the origin of protein and QOL in HD patients. Therefore, we designed a cross-sectional study to investigate the association between plant and animal protein intake and QOL in patients undergoing dialysis.

## Materials and methods

### Study population

We conducted a multi-center cross-sectional study between September 2021 and March 2022 on 264 HD patients in 5 hemodialysis centers in Isfahan, Iran. Patients were included in the study if they were ≥ 18 years old, alert, and receiving HD for at least three months. We excluded patients with incomplete questionnaires, mental disabilities, pregnant women, or if their daily energy intake was less than 800 kcal/d or above 4,200 kcal/d (28). Before being recruited for the study, each patient provided written informed consent. The Isfahan University of Medical Sciences ethical committee accepted this study’s protocol (IR.MUI.RESEARCH.REC.1399.605). This study is based on the M.S. thesis of MD.

### Dietary assessment

Dietary intake was evaluated using a validated semi-quantitative food frequency questionnaire (FFQ) that contained 168 food items, designed specifically for use in Iran (29). Previous studies showed that this FFQ could accurately reflect the dietary patterns of the Iranian population (29–32). Expert dietitians questioned patients face-to-face to complete questionnaires and describe the frequency of each food item ingested daily, weekly, monthly, or yearly in the previous year. The reported frequency for each food item was converted to a daily intake. For example, a response of “two serving/week” was converted to 0.28 servings/day. Each food serving size was converted from household measurements to grams. Nutritionist IV software was used to calculate total energy, macronutrients, and micronutrients. Protein consumption was divided into two groups: plant protein and animal protein. Legumes, nuts, seeds, grains, vegetables, and fruits were referred to as plant proteins. The animal protein group consisted of red and processed meat, poultry, eggs, fish, and dairy products.

### Quality of life assessment

Kidney Disease Quality of Life Short Form (KDQOL-SF 1/3) was used to measure QOL in HD patients. The questionnaire was administered orally by trained interviewers during the first hour of dialysis treatment. This questionnaire has 36 items and two main sections: 12 generic items that evaluate the general mental and physical status and 24 specific CKD-disease items that assess symptoms, effects, and the burden of kidney disease. The average scores for the five subscales ranged from 0–100, and higher scores represented better QOL. The validity and reliability of this questionnaire have been previously confirmed in Iranian patients with HD (33).

### Anthropometric measurements

The patient’s height was measured, without shoes, using non-elastic tape with an accuracy of 0.1 cm. After a dialysis session, dry weight was measured with the fewest clothes and without shoes with an accuracy of 0.1 kg using a calibrated digital floor scale when no signs or symptoms of hypovolemia or hypervolemia were detected (35). The body mass index (BMI) was determined by dividing dry weight by squared height.

### Assessment of other variables

Age, sex, marital and employment status, dialysis vintage, the frequency and duration of the dialysis, urea reduction ratio (URR), urea kinetics (Kt/V), and the major cause of renal failure were collected from medical records at baseline.

### Statistical analysis

The Statistical Package for Social Sciences (SPSS, version 22, Chicago, IL, United States) was used for all analyses in this study. *p* value ≤0.05 was considered statistically significant. The Kolmogorov–Smirnov test was used to determine the normality of distribution for all variables. Continuous variables are represented by means ± standard deviations, while categorical variables are represented by percentages (%). To compare categorical variables, the Chi-square test was performed, and the one-way analysis of variance (ANOVA) was utilized to evaluate continuous variables among tertiles of total, plant, and animal protein intake. Analysis of covariance (ANCOVA) was conducted to compare dietary intake after controlling for energy intake. Linear regression analysis was used to examine the association between total and type of dietary protein and QOL, with adjustment for gender, job, height, weight, URR, and total energy consumption.

## Results

### Study population

The mean ± SD age of 264 HD patients who contributed to the current study was 58.62 ± 15.26 years. Most patients were men (73.5%), married (76.20%), either retired (34.5%) or unemployed (28.4%). Most patients’ primary cause of ESRD was diabetic nephropathy (37.5%). The mean ± SD dialysis vintage was 47.11 ± 45.64 months. Moreover, the mean ± SD BMI was 24.53 ± 4.54 kg/m2. According to nutritional status, 7.7% of patients were undernourished, 49.8% had normal weight, 31.8% were overweight, and 10.7% were obese. Furthermore, the means of anthropometric characteristics, such as height and dry weight, were 164.93 ± 9.22 cm and 66.98 ± 14.64 kg, respectively. The mean of total, plant, and animal proteins intake were 66.40 ± 34.29 g/d, 34.60 ± 18.24 g/d, and 31.80 ± 22.21 g/d. Moreover, this study’s mean Q.O.L. score of H.D. patients was 59.29 ± 18.68.

### General characteristics of the patients among tertiles of the total, plant, and animal proteins intake

The general characteristics of HD patients among tertiles of the total plant and animal protein intake are presented in Table 1. As shown in Table 1, the percentage of men was significantly higher in the last tertile of total, plant, and animal proteins compared with the lower tertiles (*p* < 0.001 for all tertiles of protein intake). Furthermore, there were mostly retired subjects in the top tertile of all types of protein intake whereas most unemployed individuals were in the lowest tertile (p < 0.001 for all tertiles of protein intake). Across different tertiles of total protein intake, patients in the top tertiles had significantly higher dry weight (*p* = 0.01) and height (*p* < 0.001) compared to the patients in the first tertiles. Additionally, patients in the top tertiles of total protein intake, had significantly lower dialysis sessions (*p* = 0.02) and URR (*p* < 0.001) than the lowest tertile. Within the plant-based protein, patients in higher tertiles had significantly higher height (*p* < 0.001) and URR (*p* = 0.03) compared to the patients in the lower tertiles and those in lower tertiles had significantly higher dialysis sessions (p = 0.02) than patients in the higher tertiles. Patients in higher tertiles of animal protein had significantly higher height (p < 0.001) compared to the patients in the bottom tertile. Moreover, the major cause of ESRD in most patients in the top tertile of animal protein intake was diabetes mellitus, whereas in the lowest tertile was acute kidney injury (*p* = 0.02). No significant differences were observed regarding other characteristics throughout the tertiles of all types of protein intake.

**Table 1:** The baseline characteristics of study population across tertiles of total, plant and animal protein intake (n = 264).

Variable		Total protein		*p*		Plant protein		*p*		Animal protein		*p*
	T1 (*n* = 88)	T2 (*n* = 87)	T3 (*n* = 89)		T1 (*n* = 88)	T2 (*n* = 89)	T3 (*n* = 87)		T1 (*n* = 88)	T2 (*n* = 89)	T3 (*n* = 87)	
Age (years)	61.38 ± 14.59	57.91 ± 14.88	56.58 ± 16.02	0.09	61.47 ± 14.46	57.69 ± 15.22	56.68 ± 15.83	0.09	60.22 ± 14.70	58.83 ± 14.07	56.79 ± 16.88	0.32
Gender (male)	41(46.6)	68(78.2)	85(95.5)	**<0.001**	43(48.9)	72(80.9)	79(90.8)	**<0.001**	48(54.5)	65(73)	81(93.1)	**<0.001**
Married (%)	73(83)	70(88.6)	58(85.3)	0.58	73(83.9)	67(87)	61(85.9)	0.84	77(87.5)	67(84.8)	57(83.8)	0.79
**Job (%)**				**<0.001**				**<0.001**				**<0.001**
Self -employed	6(6.8)	10(11.5)	10(11.2)		4(4.5)	12(13.5)	10(11.5)		9(10.2)	9(10.1)	8(9.2)	
Retired	22(25)	32(36.8)	37(41.6)		23(26.1)	31(34.8)	37(42.5)		23(26.1)	30(33.7)	38(43.7)	
Unemployed	46(52.3)	24(27.6)	5(5.6)		46(52.3)	22(24.7)	7(8)		39(44.3)	27(30.3)	9(10.3)	
Other	14(15.9)	21(24.1)	37(41.6)		15(17)	24(27)	33(37.9)		17(19.3)	23(25.8)	32(36.8)	
**Cause of renal failure (%)**				0.19				0.43				**0.02**
Diabetes	37(42)	27(31)	35(39.3)		34(38.6)	32(36)	33(37.9)		35(40.2)	24(27)	40(45.5)	
HTN	32(36.4)	32(36.8)	23(25.8)		28(31.8)	36(40.4)	23(26.4)		20(23)	39(43.8)	28(31.8)	
AKI	3(3.4)	5(5.7)	2(2.2)		3(3.4)	4(4.5)	3(3.4)		3(3.4)	3(3.4)	4(4.5)	
Other	16(18.2)	23(26.4)	29(32.6)		23(26.1)	17(19.1)	28(32.2)		29(33.3)	23(25.8)	16(18.2)	
**Nutritional status (%)**				0.24				0.34				0.22
Undernourished	8(9.2)	6(7)	6(6.8)		8(9.2)	5(5.7)	7(8.1)		7(8)	5(5.7)	8(9.3)	
Normal weight	44(50.6)	45(52.3)	41(46.6)		44(50.6)	43(48.9)	43(50)		45(51.7)	45(51.1)	40(46.5)	
Overweight	22(25.3)	25(29.1)	36(40.9)		23(26.4)	28(31.8)	32(37.2)		21(24.1)	29(33)	33(38.4)	
Obese	13(14.9)	10(11.6)	5(5.7)		12(13.8)	12(13.6)	4(4.7)		14(16.1)	9(10.2)	5(5.8)	
Dry Weight (kg)	64.46 ± 14.36	65.57 ± 15.87	70.47 ± 13.91	**0.01**	63.96 ± 14.41	68.79 ± 16.43	67.85 ± 13.42	0.07	65.55 ± 15.49	66.64 ± 13.60	68.41 ± 15.60	0.44
Height (cm)	160.90 ± 8.98	164.35 ± 8.18	169.42 ± 8.50	**<0.001**	161.23 ± 8.79	165.68 ± 8.88	167.90 ± 8.83	**<0.001**	162.02 ± 8.95	165.12 ± 9.37	165.12 ± 9.37	**<0.001**
BMI (kg/m^2^)	24.84 ± 4.9	24.43 ± 5.04	24.33 ± 3.55	0.73	24.65 ± 4.94	25.06 ± 4.88	23.88 ± 3.63	0.21	24.89 ± 5.11	24.46 ± 4.29	24.26 ± 4.18	0.64
Dialysis vintage (months)	47.37 ± 39.44	50.89 ± 48.17	43.16 ± 48.97	0.54	46.33 ± 40.69	49.85 ± 46.42	45.12 ± 49.75	0.78	45.28 ± 39.34	52.39 ± 43.72	43.65 ± 53.08	0.41
Dialysis session per month	11.31 ± 1.7	10.96 ± 2.12	10.39 ± 2.92	**0.02**	11.31 ± 1.62	10.98 ± 2.35	10.35 ± 2.82	**0.02**	11.26 ± 1.92	10.88 ± 2.26	10.51 ± 2.74	0.11
URR (%)	75 ± 20	71 ± 12	67 ± 7	**<0.001**	68 ± 10	71 ± 15	74 ± 16	**0.03**	72 ± 15	72 ± 13	69 ± 14	0.46
Kt/V	1.35 ± 0.22	1.34 ± 0.23	1.29 ± 0.22	0.19	1.37 ± 0.24	1.30 ± 0.24	1.31 ± 0.20	0.05	1.35 ± 0.20	1.32 ± 0.25	1.31 ± 0.22	0.55

*p*-values for continuous variables were calculated using one-way ANOVA and Chi-square test for categorical variables. *p* values < 0.05 were considered statistically significant. Categorical and continuous variables data are presented as number (percent) and mean (SD). AKI, acute kidney injury; BMI, body mass index; HC, hip circumference HTN: hypertension; URR, urea reduction ratio; WC, waist circumference.

### Dietary intakes across tertiles of the total, plant, and animal proteins intake In HD patients

Dietary intake among tertiles of the total, plant, and animal proteins intake are shown in Table 2. All dietary protein sources significantly increased across the tertile of total protein intake (*p* < 0.05). Furthermore, total energy intake, carbohydrate, fat, SFA, cholesterol, sodium, potassium, phosphorus, calcium, vitamin B1, B2, B3, B6, B12, folic acid and vitamin C significantly increased across the tertile of total protein intake (*p* < 0.05). Moreover, patients in the top tertile of plant protein consumed higher grains, vegetables, fruits, legumes, nuts and seeds, energy, carbohydrate, sodium, potassium, phosphorus, calcium, vitamin B1, B2, B3, B6, folic acid and vitamin C compared with the lower tertiles (*p* < 0.001). In contrast, they significantly had lower intakes of red and processed meats, poultries, fishes, eggs, dairy products, fat, SFA, cholesterol and vitamin B12 (*p* < 0.05). Across different tertiles of animal protein intake, patients in the top tertiles had significantly higher consumption of red and processed meats, poultries, fishes, eggs, dairy products, energy, fat, SFA, cholesterol, sodium, potassium, phosphorus, calcium, vitamin B1, B2, B3, B6, B12, and folic acid (*p* < 0.05), and they significantly had lower intakes of grains, vegetables, fruits, legumes, nuts and seeds, carbohydrate, and vitamin C compared with the lower tertiles (*p* < 0.001).

**Table 2:** Dietary intakes across tertiles of total, plant, and animal protein intake (*n* = 264).

Variable		Total protein		*p*		Plant protein		*p*		Animal protein		*p*
	T1 (*n* = 88)	T2 (*n* = 87)	T3 (*n* = 89)		T1 (*n* = 88)	T2 (*n* = 89)	T3 (*n* = 87)		T1 (*n* = 88)	T2 (*n* = 89)	T3 (*n* = 87)	
**Food Groups**												
Grains	297 ± 215.74	336.04 ± 177.08	375 ± 225.61	<0.001	240 ± 206.45	317 ± 169.74	453 ± 214.36	<0.001	350 ± 196.98	345 ± 179.17	313 ± 205.04	<0.001
Vegetables	188 ± 168.84	241 ± 139.8	376 ± 178.62	<0.001	192 ± 168.84	274 ± 141.45	342 ± 177.08	<0.001	321 ± 159.46	273 ± 141.45	214 ± 158.44	<0.001
Fruits	288 ± 262.64	376 ± 214.36	388 ± 272.61	0.05	315 ± 253.26	377 ± 216.89	359 ± 270.28	<0.001	395 ± 234.5	362 ± 217.45	294 ± 242.32	<0.001
Legumes	27 ± 37.52	36 ± 27.96	63 ± 37.61	<0.001	27 ± 28.14	37 ± 28.29	62 ± 37.28	<0.001	35 ± 28.14	40 ± 28.29	51 ± 27.96	0.28
Nuts and Seeds	4 ± 9.38	4 ± 9.38	7 ± 9.38	<0.001	4 ± 9.38	3 ± 9.43	8 ± 9.32	0.01	5 ± 9.38	5 ± 9.43	5 ± 9.32	<0.001
Red and Processed Meat	22 ± 46.9	29 ± 37.28	42 ± 47	0.08	38 ± 46.9	35 ± 37.72	21 ± 46.6	<0.001	18 ± 37.52	25 ± 37.72	51 ± 46.78	<0.001
Poultry	11 ± 28.14	18 ± 27.96	45 ± 28.29	<0.001	26.06 ± 28.14	29 ± 28.29	20 ± 37.28	<0.001	10 ± 28.14	18 ± 28.29	46 ± 27.96	<0.001
Fish	2 ± 9.38	3 ± 9.32	10.7 ± 9.43	0.01	6 ± 9.38	5 ± 9.43	4 ± 9.32	<0.001	3 ± 9.38	3 ± 9.43	9 ± 9.32	0.01
Eggs	21 ± 18.76	18 ± 18.64	24 ± 18.86	<0.001	28 ± 18.76	19 ± 18.86	16 ± 18.64	0.01	16 ± 18.76	20 ± 18.86	27.05 ± 18.64	0.03
Dairy Products	151 ± 178.22	235 ± 149.21	322 ± 188.6	<0.001	233 ± 178.22	246 ± 150.88	229 ± 196.09	<0.001	136 ± 150.08	221.07 ± 132.02	353 ± 149.12	<0.001
**Nutrients**												
Total energy (kcal/d)	1064.30 ± 296.94	1701.73 ± 392.37	2,720 ± 101.30	<0.001	1096.18 ± 357.32	1699.77 ± 410.18	2713.20 ± 1022.95	<0.001	1220.05 ± 454.51	1729.98 ± 595.41	2,557 ± 1099.08	<0.001
Carbohydrate (g/d)	256 ± 65.66	280 ± 55.92	286 ± 66.01	0.02	238 ± 56.28	269 ± 47.15	316 ± 65.24	<0.001	281 ± 56.58	278 ± 56.28	263 ± 66	<0.001
Fat (g/d)	41 ± 28.29	58 ± 18.64	75 ± 28.14	<0.001	78 ± 28.14	60 ± 18.68	36 ± 27.96	<0.001	53 ± 27.96	57.04 ± 28.38	64 ± 28.14	0.04
SFA (g/d)	17 ± 0.09	19 ± 0.09	20 ± 0.09	<0.001	21 ± 0.09	19 ± 0.09	16 ± 0.09	<0.001	17 ± 0.09	18 ± 0.09	20 ± 0.09	<0.001
Cholesterol (mg/d)	171 ± 121.94	183 ± 93.2	251 ± 122.59	<0.001	231 ± 112.56	202 ± 94.3	174 ± 121.16	0.03	147 ± 103.93	188 ± 94.55	273 ± 102.52	<0.001
Sodium (mg/d)	3,260 ± 1842.4	3,263 ± 1453.92	3,397 ± 1857.71	<0.001	2,981 ± 1688.4	3,123 ± 1433.36	3,826 ± 1789.44	0.01	3,089 ± 1640.32	3,383 ± 1442.79	3,447 ± 1594.6	<0.001
Potassium (mg/d)	2,843 ± 1369.48	3,493 ± 1137.04	4,856 ± 1442.79	<0.001	2,859 ± 1360.1	3,607 ± 1150.46	4,755 ± 1435.28	<0.001	3,281 ± 1360.1	3,748 ± 1235.33	4,185 ± 1407.32	0.001
Phosphorus (mg/d)	869 ± 290.78	1,122 ± 242.32	1,539 ± 311.19	<0.001	988.06 ± 337.68	1,177 ± 282.9	1,372 ± 363.946	<0.001	951 ± 281.4	1,146 ± 264.22	1,441 ± 298.24	<0.001
Calcium (mg/d)	743 ± 543.94	1,082 ± 438.04	1,562 ± 556.37	<0.001	832 ± 544.04	1,072 ± 452.64	1,494 ± 568.52	<0.001	874 ± 515.9	1,162 ± 462.07	1,359 ± 531.24	<0.001
Vitamin B1 (mg/d)	1 ± 0.45	1 ± 0.38	1 ± 0.48	<0.001	1 ± 0.43	1 ± 0.35	1 ± 0.45	<0.001	1 ± 0.44	2 ± 0.39	3 ± 0.45	0.04
Vitamin B2 (mg/d)	1 ± 0.58	1 ± 0.48	2 ± 0.52	<0.001	1 ± 0.63	1 ± 0.53	1 ± 0.67	<0.001	1 ± 0.55	1 ± 0.49	2.02 ± 0.56	<0.001
Vitamin B3 (mg/d)	13 ± 0.09	16 ± 0.09	24 ± 0.09	<0.001	14 ± 0.09	18 ± 0.09	21 ± 0.09	<0.001	15 ± 0.09	17 ± 0.09	21 ± 0.09	<0.001
Vitamin B6 (mg/d)	1 ± 0.45	1 ± 0.38	2 ± 0.48	<0.001	1 ± 0.53	1 ± 0.43	2.07 ± 0.54	<0.001	1 ± 0.46	1 ± 0.42	2.07 ± 0.48	<0.001
Folic Acid (mg/d)	265 ± 140.7	328 ± 121.16	448 ± 150.88	<0.001	235 ± 131.94	328 ± 92.52	482 ± 122.02	<0.001	317 ± 140.7	358 ± 122.59	368 ± 139.8	0.049
Vitamin B12 (μg/d)	2	2	4	<0.001	3	3	2	<0.001	1	2	4	<0.001
Vitamin C (mg/d)	142 ± 84.42	142 ± 74.56	147 ± 94.3	<0.001	131 ± 84.42	148 ± 66.01	157 ± 93.2	<0.001	153 ± 75.04	148 ± 66.01	135 ± 83.88	<0.001

*p*-values were calculated using ANCOVA test adjusted for energy intake. *p* values < 0.05 were considered statistically significant. Variables are presented as the means ± standard deviations. SFA, saturated fatty acid.

### Association between QOL and different types of dietary protein intake In HD patients

The results of linear regression between the QOL and different types of dietary protein intake among HD patients are presented in Table 3. In the unadjusted model, significant positive associations were seen between total (*β* = 0.16; *p* < 0.001), plant (*β* = 0.32; *p* < 0.001), and animal (*β* = 0.16; *p* < 0.001) protein intake and QOL. Similarly, in model 2 after adjusting for sex, job, height, weight and URR, significant positive associations were seen between total (*β* = 0.14; *p* < 0.001), plant (*β* = 0.29; p < 0.001), and animal (*β* = 0.12; *p* = 0.01) protein intake and QOL. Furthermore, in model 3, after adjusting for model 2 confounding variables in addition to total energy intake significant positive associations were seen just between total (*β* = 0.12; *p* = 0.03) and plant (*β* = 0.26; *p* < 0.001) protein intake and QOL. However, in model 3, the association between animal protein intake and QOL was not significant (*β* = 0.03; *p* = 0.60).

**Table 3:** The association between quality of life and different types of dietary protein intake in hemodialysis patients (*n* = 264).

Models	Total protein		Plant protein		Animal protein	
	*β* (95% CI)	*p*	*β* (95% CI)	*p*	*β* (95% CI)	*p*
Model 1	0.16 (0.09,0.23)	<0.001	0.32 (0.20,0.44)	<0.001	0.16 (0.06,0.26)	<0.001
Model 2	0.14 (0.07,0.21)	<0.001	0.29 (0.16,0.42)	<0.001	0.13 (0.02,0.23)	0.01
Model 3	0.12 (0.06,0.23)	0.03	0.26 (0.07,0.45)	<0.001	0.03 (−0.09,0.16)	0.60

*p* values were obtained from linear regression. Beta coefficients and their respective confidence intervals 95% (95% CI) were reported.

*p* values < 0.05 were considered as statistically significant.

Model 1: unadjusted. Model 2: adjusted for sex, job, height, weight and URR. Model 3: adjusted for sex, job, height, weight, URR, and total energy intake.

## Discussion

Nutritional status, especially dietary protein intake, is an essential factor that influences the QOL of HD patients. However, few studies are available on the association of dietary protein with QOL in these patients (34–36). In addition to the quantity of protein, the source of protein may be an influential factor in the complications and QOL of HD patients (21, 37). This study is the first to evaluate the association between the source of protein and QOL in HD patients. We observed that higher consumption of total and plant proteins were associated with better QOL in HD patients, while there was no significant association between animal protein intake and QOL.

There are limited studies on the association between total dietary protein and QOL in HD patients (6, 36, 38). In line with our study, Shahrin et al. showed a significant positive correlation between protein intake and QOL in HD patients (6). However, Sharin’s study was conducted in Malaysia and their patients were older than our study. Moreover, the mean of total dietary protein intake in their study was lower than our study. Two other cross-sectional studies revealed that low dietary protein intake, as indicated by low serum albumin levels, is independently associated with poor QOL among HD patients (36, 39). Possible mechanisms may include the important role of dietary protein intake in the catabolic process in HD patients, which helps prevent muscle wasting and decrease the risk of infection (40). Furthermore, low protein consumption may cause anemia, weakness, and fatigue, directly affecting QOL’s physical and mental components (8, 41). In contrast, Yusop et al. found that lower protein intake was associated with better QOL in HD patients. This study also revealed that patients who did not achieve the protein intake recommendation still had good QOL scores and better BMI status (42). The discrepancy between the results of our study and those reported by Yusop et al. could be due to the different methodologies used to assess dietary intakes. Yusop et al. used 24-h diet recall, whereas we used the FFQ, which evaluated dietary intakes over the past year and may be more accurate.

In addition to the total protein intake, the source of protein can also affect kidney function and complications in HD patients (23, 24, 43, 44). In this study, we found that a higher intake of plant protein is associated with better QOL. Considering that there has been no study on the association of protein sources with QOL in these patients, the mechanism underlying this relationship has not been adequately addressed. However, various studies have been conducted on the source of protein intake and the complications of HD patients, which can affect the mental and physical dimensions of QOL in these patients. Cardiovascular disease (CVD) is one of the most common complications that crucially affect QOL and is the first cause of death in HD patients (45). Plant-based diets and increased protein intake from plant sources can reduce the risk of CVD in these patients (46). Plant-based proteins are rich in polyphenols and fiber, which significantly lower inflammatory factors, total and low density lipoprotein (LDL) cholesterol (47). Furthermore, patients who consume higher levels of plant protein have higher serum levels of threonine and histidine amino acids. The high level of these amino acids is associated with better blood pressure control. In contrast, red and processed meat are rich in SFA and sodium, increasing inflammatory markers, cholesterol, and serum concentrations of alanine and methionine. These factors are associated with hypertension and increased CVD complications in patients (48, 49). Moreover, higher plant protein intake can reduce the dietary acid load (DAL), while higher consumption of animal protein yields a higher DAL, which increases acidosis and decreases kidney function (50). A cross-sectional study revealed that higher DAL was associated with depression and sleep disorders. Therefore, plant-based proteins may have favorable effects on the mental health and QOL of patients (51). Further, uremic toxins accumulate in HD patients and cause various adverse effects, such as increased inflammation, oxidative stress, insulin resistance, increased CVD risk, and suppression of appetite (52). Plant-based proteins reduce the production of uremic toxins by modulating gut microbiota, improving patients’ conditions (53). Also, increasing the intake of plant protein may be associated with better appetite and calorie intake, which decrease the risk of malnutrition and ultimately improve QOL (54). Regardless of the benefits of plant-based proteins, one of the biggest concerns of using these sources in HD patients is the risk for hyperkalemia. However, the studies showed that high intake of these sources, despite their higher potassium contents, has not been shown to cause hyperkalemia in these patients (55). This may be because they are high in fiber, facilitating potassium excretion, whereas animal-based proteins worsen constipation and increase the risk of hyperkalemia (23). Another concern about plant-based protein is the risk for hyperphosphatemia. Although some plant-based protein like legumes, seeds and nuts have a high phosphate content, phosphates in these sources are stored in phytate form, which are difficult to digest, and only 10–30% of them are absorbed. In contrast, phosphates in animal-based protein have much higher bioavailability, and 40–60% of them are absorbed in the human gut (56, 57). A cross-sectional study showed that HD patients on vegetarian diets had significantly lower serum phosphate levels than non-vegetarians (58). Despite these considerations, hyperkalemia and hyperphosphatemia can have severe health consequences. Therefore, plant-based protein should still be consumed with caution in HD patients. Because of the benefits of plant-based proteins on the different complications of HD patients and the effects of these complications on the QOL, there may be a positive association between the plant protein and the QOL of these patients.

While this is the first study that has examined the association between the source of protein and QOL among HD patients, there are several limitations. The main limitation of this study was the absence of laboratory outcomes. Including biochemical assessments would have allowed for a more precise investigation of this association. Moreover, since this was a cross-sectional study, causality cannot be established. Furthermore, although a validated FFQ was used in this study, there may be some measurement errors and recall biases for a dietary intake assessment. Finally, many factors may have influenced the QOL that the researcher could not control.

## Conclusion

In conclusion, our findings indicate that higher consumption of plant protein than animal protein is associated with better QOL in HD patients. However, clinical trials and cohort studies are required to make a clear conclusion.

## Data availability statement

The raw data supporting the conclusions of this article will be made available by the authors, without undue reservation.

## Ethics statement

The studies involving humans were approved by Isfahan University of Medical Sciences (Code: IR.MUI.RESEARCH.REC.1399.605). The studies were conducted in accordance with the local legislation and institutional requirements. The participants provided their written informed consent to participate in this study.

## Author contributions

MR designed the study. MD collected data and entered data into the software. MR analyzed data. MD wrote the initial manuscript. S-AK revised the manuscript. All authors contributed to the article and approved the submitted version.

## Funding

This study was supported by the Isfahan University of Medical sciences (grant number: 199466). The funders had no role in study design; collection, analysis, and interpretation of data; writing of the report; the decision to submit the report for publication.

## Conflict of interest

The authors declare that the research was conducted in the absence of any commercial or financial relationships that could be construed as a potential conflict of interest.

## Publisher’s note

All claims expressed in this article are solely those of the authors and do not necessarily represent those of their affiliated organizations, or those of the publisher, the editors and the reviewers. Any product that may be evaluated in this article, or claim that may be made by its manufacturer, is not guaranteed or endorsed by the publisher.
